# Prior authorization and utilization management for post-acute home health in Medicare Advantage: the motivations, players, processes, unique challenges, and impacts on patient care

**DOI:** 10.1093/haschl/qxaf020

**Published:** 2025-02-04

**Authors:** Kali S Thomas, Marguerite Daus, Christine Jones, Jennifer N Bunker, Jamie M Smith, Jeffrey Marr, Emily A Gadbois

**Affiliations:** School of Nursing, Johns Hopkins University, 525 N Wolfe St, Baltimore, MD 21205, USA; School of Medicine, University of Colorado, Aurora, CO 80045, USA; School of Medicine, University of Colorado, Aurora, CO 80045, USA; School of Nursing, Johns Hopkins University, 525 N Wolfe St, Baltimore, MD 21205, USA; School of Nursing, Widener University, Chester, PA 19013, USA; Bloomberg School of Public Health, Johns Hopkins University, Baltimore, MD 21205, USA; School of Public Health, Brown University, Providence, RI 02903, USA

**Keywords:** post-acute care, home health, managed care, prior authorization, utilization management, Medicare

## Abstract

In 2024, 90% of Medicare Advantage (MA) enrollees were in a plan that required prior authorization of home health care. We conducted semi-structured interviews with 44 leaders of MA plans, post-acute care (PAC) management companies, and home health agencies (HHAs) across the country to understand their experiences with prior authorization and utilization management (UM) of post-acute home health care. Our analysis of these interviews revealed that representatives of MA plans and PAC management companies report varying motives and approaches to prior authorization for post-acute home health care, resulting in varied experiences for HHAs. Both MA plan and HHA representatives view prior authorization and UM of post-acute home health as burdensome, and each have taken distinct approaches to manage the process but have conflicting views on the utility of these approaches. Home health agency representatives report that prior authorization and UM requirements impact access to care, the way that care is delivered, and ultimately patients' experiences. Our findings warrant additional research and policy attention so that MA plans' UM techniques do not unintentionally cause patient harm, particularly among vulnerable Medicare enrollees in need of post-acute home health care.

## Introduction

As of 2024, more than 50% of all Medicare beneficiaries were enrolled in a Medicare Advantage (MA) plan.^1^ One very common approach used by MA plans to control healthcare costs and ensure appropriate use of services is through prior authorization: a process that requires review and approval of services or treatments for patients before they are rendered. Research on the use of prior authorizations in the MA program suggests that it has increased dramatically in the last several years, with 99% of MA enrollees in a plan with a prior authorization requirement in 2024.^2^ While prior authorization is one mechanism plans use to avoid unnecessary care, it has been reported to lead to delays in care and create barriers to timely access to necessary services.^3^ For example, a 2022 OIG report found that MA plans improperly denied or delayed care by requiring prior authorization for services such as imaging and post-acute care provided in skilled nursing facilities.^4^ These denials and delays in care can disproportionately impact people who need more complex and frequent care, including the vulnerable group of patients leaving the hospital in need of post-acute care (PAC).

Post-acute care can vary in intensity and setting depending on each patient's needs. Post-acute care is often delivered in institutional settings;^5^ however, as healthcare entities look to both meet patients' preferences to be home and reduce costs associated with institutional care, PAC is increasingly shifting toward care provided in patients' homes by home health agencies (HHAs).^6^ Post-acute home health care provided by Medicare-certified HHAs includes skilled nursing services and therapies that aim to help patients regain or maintain their health and function.

While prior authorization for institutional PAC has recently faced increased scrutiny,^7^ less is known about prior authorization for post-acute home health care. There is a need to understand this utilization management (UM) practice in home health given 90% of MA beneficiaries are enrolled in a plan requiring prior authorization for home health.^2^ Additionally, prior studies^8-10^ have found that patients enrolled in MA were less likely to receive post-acute home health care: those with MA who do receive PAC home health have shorter lengths of stay and fewer nursing and therapy visits than patients leaving the hospital enrolled in traditional Medicare, resulting in worse functional status outcomes.^11^ These studies are quantitative (except 1 study that included a qualitative component with 9 informal interviews^8^), and thus unable to provide insights into the underlying mechanisms by which these MA differences occur. Our study aims to fill this gap through systematic qualitative interviews and analyses; therefore, the objective of this study was to understand the perspectives of MA plans, PAC management companies, and HHAs on processes that MA plans use for prior authorization and UM for post-acute home health care.

## Methods

We conducted semi-structured interviews with participants from MA plans, PAC management companies (contracted by MA companies to interface with care providers to manage services and payments, and sometimes referred to as “convenors”), and HHAs. Participants were selected using purposive and snowball sampling. We focused on 6 US markets with moderate-to-high MA penetration and at least 3 MA plans serving 10% or more of Medicare beneficiaries. Recruitment of MA plan representatives was done via professional networks, LinkedIn, and web searches, followed by PAC management companies and HHA recruitment.

Two semi-structured interview guides were developed and piloted with industry experts. Interviews, conducted by phone or video between March 2023 and June 2024, lasted about an hour and were recorded with participants' consent. Recruitment continued until saturation was achieved.^12^

Interviews were transcribed and analyzed using modified content analysis.^13^ Preliminary coding schemes were developed and refined through an iterative process. At least 2 team members coded each transcript, reconciled codes and summarized findings from each interview. Weekly team meetings were used to discuss emerging themes; we kept a comprehensive audit trail to document team impressions and decisions.^14^ Data were managed in NVivo 1.7.1. (QSR International). The project was not classified as human subjects research by Brown University’s Institutional Review Board.

### Limitations

This study has limitations to note. Because this is a qualitative study that includes a sample of MA plans, PAC management companies, and HHAs obtained through purposive and snowball sampling,^15^ results may not be generalizable. While we attempted to select plans and HHAs that varied in size, age, quality, and region, participants may differ in their perspectives and experiences from others. Additionally, social desirability bias could affect the openness of participants about challenges.^15^ Nonetheless, our study is the only study to our knowledge that has systematically investigated the experience with prior authorization and UM from the perspective of multiple players through systematic qualitative interviews and analyses.

## Results

Interviews were conducted with 3 groups of participants: MA plan, PAC management company, and HHA leaders. A total of 18 individuals from 14 MA plans were interviewed, with positions including presidents or chief executive officers, chief medical officers, chief development officers, and various vice president roles. Participating plans were national or regional in scope, had a range of publicly reported CMS quality star ratings, and varied in size (see Appendix Table S1). Participating plans collectively enrolled more than 20 million total MA members in 2023 (>62.4% of the total MA market). We also interviewed 1 director, 3 vice presidents, and 1 chief medical officer from 5 PAC management companies. Finally, we interviewed 21 individuals from 19 HHAs. Positions included presidents, chief executive officers, administrators, chief operating officers, and directors. Home health agencies were national or regional in scope, had a range of publicly reported CMS quality of care star ratings, ranged in age, and ranged in the number of new patients seen in 2020 (see Appendix Table S2). In the following paragraphs, we discuss 4 themes identified across the 44 interviews.

### Theme 1: MA plan and PAC management company representatives report varying motives and approaches for prior authorization and UM for post-acute home health, resulting in varied experiences for HHAs

Medicare Advantage plan representatives report varying motives for applying prior authorization and UM for post-acute home health care (Theme 1a). While some plan and PAC management companies report that they require prior authorization to “reduce[e] costs” (MA 12) and address “bad actors” (CO 5), MA plans also described using these approaches to share information, “be supportive… check in… and close gaps” (MA 1). However, some MA plan representatives shared that regardless of the rationale for applying prior authorization requirements, they do not believe that prior authorization is the appropriate way to do UM for post-acute home health. See Table 1 for representative quotes.

**Table 1.** Theme 1.

Theme 1a: MA plan representatives report varying motives for requiring prior authorization and utilization management for post-acute home health		
Plan reports prior authorizations control spending	Well, so a lot of the goals are reducing costs, but then also reducing burden. Right? So, you don’t want to have to have a prior authorization for things that are low hanging fruit. You want to make sure that if you need a prior authorization, it's for a good reason. Now, post-acute, that is something that generally requires a prior authorization, because it's a higher cost thing for both the member and the plan. So, that is definitely something that requires a prior authorization.	MA 12
Post-acute care management company says that UM protects against “bad actors”	That [re-authorization] is one of those things that's pretty burdensome for providers. There's just certain things you can’t avoid because there are too many bad actors out there.	CO 5
Plan reports that authorizations add administrative hassle and aren’t the way to do UM	The administrative hassles overweigh the kind of utilization concerns, because they know that the delivery network will be appropriate and they monitor it in a different fashion than through authorizations. Authorizations are not a good way of managing utilization. That's a crude way of doing it. I don’t like that. I try to avoid it.	MA 14
Plan does full authorization and thinking of changing it	We do full on UM. I am actually not supportive of that… I’ve come into a few health plans where we were doing it and I stopped doing it…I think home healthcare is sort of like physical therapy or behavioral health. There's a certain number of visits you should be authorized. You should just let it happen… there is also another point where it's abusive. Is it 8 visits, 10 visits, 12 visits? I don’t know. I haven’t decided. But there is probably a visit level before, which you should not be doing any authorizations. You should just do a notification and then you can authorize after the fact. I think that's where there's a lot of variability. We do authorize and we are actively discussing changing that up to a certain amount.	MA 9
Plan reports that authorizations and information sharing are a way to support HHAs, “close loops and gaps”, not “auditing”	So, it's really, they write the care treatment plan when they assess the individual… It's less about prior authorization, it's more about just connecting to make sure we’re all in agreement… Because we do know there's shortages and so we will utilize our staff or our care managers or to follow up too, just to make sure that things are copacetic and we see that as a value add not to be critical, but to be supportive, to be an adjunct or a complement to what's happening. So, it's not worth so much that we’re auditing what that is. We don’t really look at it that way. It's more just that check in that follow up just to close any loops and gaps.	MA 1

Theme 1b: Plans vary in their approaches to applying prior authorization and utilization management for post-acute home care, resulting in varying experiences for home health agencies		
Plan auto approves HHA for 15 days; but that varies even within an organization	We auto approve to a certain amount, 15 visits, but there are other caveats to that depending geographically like Florida, super convoluted and as well as on the West Coast, we also have a lot of risk delegation too. And then they’re auto approved, and then if they need more visits after that, then the agency has to write back and make another request for additional visits and what the criteria are needed. That goes over to a team that processes the request.	MA 10
Plan approves 30 visits without any review; then very light touch	We, as a plan, give 30 units right up front without any concurrent review, so that agencies just have to notify us that they have taken on one of our members and it just kind of gets plugged into the system and pays the first 30 units. And then we do, I would call very light concurrent review after that. We love home care, we want our members home, we want our members to get skilled home care.	MA 4
Post-acute care management company does not require prior authorization, but does UM	The home health agencies get a referral and they can immediately see the patient. I mean, the last thing that we would want them to do is to delay care because they’re waiting for an authorization for home care. So that initial home care visit is always approved. And then what we ask is we ask for the OASIS assessment, so the structured OASIS after the assessment is done, and then we use that assessment to basically do all of our utilization management. When we collect the OASIS, we run algorithms, the proprietary algorithms that we’ve built over the last 10 years, and we tell them how many visits they can have if they’re a fee for service. Now that we’ve moved to episodic, I mean, as long as they’re home bound and approved and they submit the OASIS and we can do all the clinical algorithms that we have, whether or not they do six visits or 15 visits, they’re on the hook to get better care for my members. And so, I’ve pushed the decision of how many visits they should do to the clinicians.	CO 2
Post-acute care management company does not require prior authorization, but does require reauthorization	What we generally try to do is not be a barrier to any sort of timely start of care. For obvious reasons. You have a patient coming out of facility, you don’t want them sitting there in their home without care… So, we’re generally auto adjudicating, the initial authorizations at a pretty high rate. We want to make sure that our providers don’t have any barriers, that they’re getting in there. They’re providing care, they’re assessing the member, they’re performing that OASIS^a^, they’re working with the physician to come up with a point of care. And then generally we ask around day 30 for that provider to come back to us and request what we call a reauthorization.	CO 4
Post-acute care management company does not require prior authorization, but does UM	Hey, if they need home health, we’re happy to start it up… We approve basically a hundred percent of any referral that comes out of a facility because if you’re coming out of a facility, then you probably need skilled home health and you qualify for it… And so, it's more about the ongoing service needs that are also reviewed by a UM nurse… And so, what happens is the moment we receive that referral or prior auth request, a person is reviewing that and assigning it to a clinical category, and then we have predefined visit sets based on those clinical categories. And it's sort of like a get-in-the-door authorization is what we used to call it, but now it's typically enough for the first three weeks, potentially even first 30 days depending on the acuity level of that member… We ask that before the visits end that they submit a reauthorization request to us and to a point where if it's clear that medical necessity is there and you can prove it in your clinical notes, send that referral in. You could have 10 visits left, but if it's clear that you’re going to need those 10 and some, go ahead and send that in and our approval rate is pretty high. It's like in the 80s in those instances.	CO 5
Post-acute care management company does not require UM, including prior authorization, because home health initiation is complex enough	Home health is different because there's no prior auth in home health… The doctor says they need home health, they go out, they get their home health provider, the home health provider sees them without even having any kind of involvement with us. That was a deliberate design decision. We didn’t want to get in the way of home health starts. That's a big issue. You don’t want to slow down getting a home health into the patient's home because typically, you want to get it there as quick as you possibly can. That always improves results… How do you get from discharge to start of home health that is rocky and that's one area we’re trying to solve for.… Let's not add any more complexity and require an auth on anything because it's already complex enough and falls down often enough that we want to stay out of that.	CO 3
Plan does not require prior authorization to speed up getting home care in home	The one thing with those patients coming right out of the hospital is those are already pre authorized, so they don’t have to go through the utilization management department, so we stopped that so that there was no delay in getting the patient that care out of the hospital, and so they don’t have to go through the UM, and so they can start that care right away	MA 7
HHA reports variation by plan, according to size	[Name of Regional Plan], we don’t have to wait for authorization. We can just do what we have to do and they get out of our way. And then you have [Name of Large National Plan] who's, “Well, we’re going to know every move you make before you make it,” and it delays things.	HHA 9
HHA reports variation by plan, even worse for plans with post-acute care management companies	It really, really, really depends, by plans. So, some of our, and I am going to knock on wood, I am luckier than a lot, but some of my MA contracts, we negotiated PDGM Medicare rates, so the authorization process is like it's non-existent, it's treated just Medicare, easy-peasy, lemon squeezy. For some plans, they just are very different. Some of them we have to get an authorization to start the care, but once we’ve started the care, then we’re good to go. Some of them make us authorize for a month at a time. Some of them are making us authorize by visit. And then if you are stuck dealing with a convenor, you literally are begging and pleading for every single visit and we will tell them we’re going to need 10 visits and they authorize it one at a time.	HHA 19
HHA reports range in needing authorization prior to assessment vs after assessment	Depending on the managed care plan, we might have to get authorization before we even start the care. And with some of them, we get authorization for that initial visit and then send a plan of care in and then they decide how much more care we can provide to the patient. So, there's two ways. Sometimes we automatically can go in that first visit and sometimes we have to wait until we have authorization for the first visit.	HHA 4

HHA = Home Health Agency, MA = Medicare Advantage Plan, CO = Post-Acute Care Management Company.

^a^The Outcome and Assessment Information Set (OASIS) is a standardized assessment required by 42 CFR 484.55 as part of the home health agencies' Conditions of Participation, completed by a registered nurse (RN) or qualifying therapist (eg, physical, occupational, or speech therapist) to assess patient needs and verify eligibility for the Medicare home health benefit (ie, homebound status and need for skilled services).

Source: interviews with participants.

Medicare Advantage plan and PAC management company representatives also revealed that plans take very different approaches to prior authorization and UM for post-acute home health (Theme 1b). For example, some plans and PAC management companies auto-approve post-acute skilled home health in an effort not to slow down the process—or further complicate an already complicated process of getting services in the home. Others report that doing a “light touch review” (MA 4) to make sure patients meet criteria for post-acute home health and continuation of services is necessary. We also heard from MA plans that require detailed and complete information before authorizing post-acute home health.

Home health agencies echo this reported variation, sharing that plans are very different in their processes for UM, including if they require prior authorization at all. Home health agencies describe some plans that allow the HHA to conduct the initial assessment, determine a plan of care and then “rubber stamping it” (HH 5), whereas other plans are described as requiring HHAs to ask “mother may I” (HH 5) and wanting to “know every move you make before you make it” (HH 9). This variation is extended to the number and types of visits authorized with each request (see Table 1).

### Theme 2: Both MA plans and HHA representatives view prior authorization and UM of post-acute home health as burdensome; each have taken distinct approaches to manage the process but have conflicting views on the utility of these approaches

Our interviews revealed the burdens that home health prior authorizations and UM place on MA plans and HHAs. Recognizing the need to ease those burdens, some common approaches discussed by both MA plans and HHA representatives included hiring additional staff, training clinicians, placing staff in hospitals to help with prior authorization requirements, or creating departments solely responsible for UM functions (Theme 2a; see Table 2).

**Table 2.** Theme 2.

Theme 2a. Dedicated, trained staff for UM functions		
Plan has staff in hospital to help with authorization	We require an authorization. With our larger hospitals, we have a nurse that we actually embed at the hospital or put in the hospital for assistance with post-acute care and discharge planning… They’ll pick it up and put the authorization in the system for the services… we believe the patient should get to the right setting as quickly as possible. That's always been our goal is to get them to that most appropriate setting of care as quickly as possible.	MA 6
Plan has team to help with authorization	Our transition of care team works very closely so there's no delay in having those services authorized. Because our number one goal is, always if somebody can get back home, that will be their first stop.	MA 3
HHA trains clinicians	We spend a lot of time with the documentation, training clinicians so that they understand not only how to document, but how to assess, how to understand what's appropriate for home health, what makes somebody homebound, what makes somebody appropriate for discharge. Really, we focus on these things all the time. It's constant education.	HHA 7
HHA has dedicated team to do UM functions	We have a whole team of people who will sit there just managing authorizations and documentation back and forth, it's like pulling teeth.	HHA 5
HHA hired third-party company to offload the administrative burden	We did this with our own people for a while, and it was just a nightmare. So, we’ve contracted out with a company, one of the best in the industry, that does our eligibility, our authorization, and they do our billing for us as well.	HHA 13

Theme 2b. Post-acute care management companies and delegated providers		
Plan uses vendors to manage the entire process	In our case, the hospital discharge planner would contact the number for our vendor and we would let them know the geography… They make sure that there's a home health agency that can get there usually the day of, ideally if not the next day, that they’re going to be able to provide all the modalities that are going to be needed… They make sure that the geography's okay, that there's availability, adequate staffing and that they have the modalities and then off the go. They do a prior authorization. For us, it is a full UM function.	MA 9
Plan uses delegated providers to manage the administrative burden on behalf of the plan	Some organizations are delegated to make that decision. They won’t ask for an authorization from us, they’ll do it on our behalf, which works well, because then there's no back and forth that needs to happen… Because administrative work has its cost as well, so on the one side, you’re trying to make sure that there's appropriate utilization, but at the same time, you don’t want to burden yourself with overly administrative tasks as well.	MA 14
Plan delegates UM, but has oversight	We contract with large medical groups and as part of that contract, we pay them a capitated fee per member per month… we delegate a utilization management function to them. And part of that utilization function is for them to make authorization decision, either approval or denial for inpatient stay, skilled nursing facility stay, [durable medical equipment], and also home health…From time to time we will audit them to make sure that they do it correctly and in compliance because we get audited by a regulatory agency to make sure that we are doing it correctly. So, we delegate, but we also have oversight. We are ultimately responsible for the group to be in full compliance with the regulatory requirement.	MA 13
HHA describes PAC management companies' roles as managing managed care	We say it's managed, managed care because it's the company managing the managed care… It's taken another level of management and reviewing everything.	HHA 7
HHA reports that working with PAC management companies (conveners) increases administrative overhead	The thing that I want to talk about is the pebble in my shoe right now, and that is lovely people that have come into the market called convenors… So, what they do is they actually go in and pull an exclusive contract with the MA plans. So, the MAs typically go capitated with [name of PAC management company] then they don’t actually do anything other than siphon money off the top. They go out and they contract with all sorts of agencies in the area at these ridiculously low per-visit rates. And then they do utilization management on top of it and limit the number of visits, which I think is probably where we’re going to end up going with this… They tell that they’re the greatest thing since sliced bread and they help the agencies and they lower the administrative burden. I actually have documented evidence because we’ve done studies to report back to the system lies and untruths all of it. It increases my overhead admin to deal with these yahoos by 25%. And then they’re paying these ridiculously hideous rates that I lose money even if it was the same administrative overhead.	HHA 19
HHA reports that PAC management companies slow down authorization process	[PAC management companies] It slows down discharges, slows down the auth process. It does decrease the patient's choice because of those issues. [Name of PAC management company] is very well known as being very difficult to work with, both in getting auths, both in making referrals. Even for our home care agency, we try not to take [Name of post-acute care management company]. Of the authorization that is the oldest on our books right now, it's [Name of post-acute care management company], it's us having to go back multiple times, continue sending in the claim, “Oh, it's under review.” “Oh, we’ve lost it.” They’re terrible to work with.	HHA 8
HHA describes as working with a PAC management company as “not even worth it”	[Name of plan] uses [name of PAC management company], the requirements to get service for a patient and get us out, it's really not even worth it to us because it's so much involved and it's every week or so many visits, you’re going to do it again and again and again, and it's really difficult.	HHA 9
HHA reports that PAC management companies are learning	I am not as grumbly as I used to be about it. They were a nightmare for many, many years where it was, and they would even say to us, we have no incentive for quality outcomes. Our job is to reduce cost. So, it's manage utilization really, really, really tightly…I think some of them realized that was not a long-term solution or people wouldn’t want to contract with them. So, we’ve seen some softening… They were very transactional, very pulling teeth. But now they’ve warmed up to, let's think about value. Let's think about quality. Let's try something different. So, it's changing for sure.	HHA 5

Theme 2c. Portals		
Plan uses portal that includes other data so they can fully understand care	[Name of software] is a very great platform that has a lot of information that is very essential to the care coordinators to know. We can look at utilization management information, claims data and other information, sometimes documentation from others that are also using [name of software], so that we can get sort of a whole picture of individuals.	MA 8
Plan uses portal to speed up approval process	We have just rolled out a provider portal that allows for the authorization for home health to be put in on the portal without calling us. We are just now rolling that out. In the past, there had to either be a fax to us for us to enter the authorization for our phone call…We’re trying to speed that up through the portal.	MA 6
Plan electronically authorizes post-acute home health care through portal; provides real-time data on members	We have a portal where we ask the home health to upload the OASIS, and then we’re sort of electronically authorizing, because we recognized that we were sort of authorizing like 95% of our home health requests anyway. Unless there's something out of the ordinary, they would sort of get an automatic auth and we would also get some clinical data real time on the members because they’re uploading that information into the portal.	MA 2
PAC management company uses portal with algorithm to speed up approval process	Our process used to be a home care agency would go into our provider portal. They’d fill out a 45-minute survey. We would get the survey. They would fax in all of their clinical documentation, and then a nurse would look at the clinical survey that they did and then the documentation, take one to three days and then get back to the home care agency and tell them that they were approved… Now we’re just collecting the OASIS, we run algorithms on that and within two minutes they get approval right in the system… Instead of spending all those nurses' times saying whether or not a patient should get home care, we’re using those same nurses to say, how do you manage the chronic conditions of these members?	CO 1
Plan doesn’t use portal but is looking to speed up and provide insight into approval process	We are looking for technology to help better support and also just better measure the length of time that it takes for home health agencies to pick up the cases, as well as then the length of time that it actually takes them to have boots on the ground in the home. It can be a little bit of black box in between.	MA 3
Plan uses portal that ties to criteria that determines appropriateness for continued services	They [HHAs] just go into our portal and they plug the 30 units in themselves, and it spits out an approval number and they can use that to bill us. And then they have to go back into the portal and request additional time after that, so we usually hear from them around unit 25 being used. They’re like, “Oh, we’re running out and we still need 30 more.” And then they go in and request. And at that time, they fill out InterQual, which is like an algorithm, and it basically, it's check marks for the home care agency. “Is this member in fact home-bound? Are they under the direction of a physician? Is the goal short term? And do they have active goals and care planning?”. So long as that criterion is met and we see the supporting documents, then we approve the additional units.	MA 4
MA plan uses portal to send information to clinical team, but inconsistency in review is a problem	So, it's all through an affiliate link, so it's all computer based. They send those modification requests. It goes straight to our department for our nurses to review…I think there's inconsistency across the team. Some I don’t think do a thorough enough review of the patient's chart and they try to go too fast. That's my perception because you’ll see one nurse will deny a patient for home health and the exact same type of diagnosis will get approved for a large number of visits, and so I think there's inconsistency across the team, and that's an issue, and we have to address that myself and my program manager because we get the feedback, whether it's from the agencies or it's from the care teams, and so I would say that's a big issue and challenge, and then just the timeliness. There seems to be always issues with staffing, and the UM team is consistently out of the criteria as far as authorizing new starts of care, and then also authorizing the modifications that get put in.	MA 7
HHA reports having 4 different portals	Yeah, there's many different, I don’t know if those are called clearing houses or portals. Let's just say portals for lack of a better word. Depending on the insurance, you have to go into that payer's portal and request authorization for visits or submit company's NPI number, as well as the member ID and establish the episode. Most of the time, it requires uploading our OASIS documentation and our plan of care and it really, really just depends on the payer which portal you’re going into, so I think we have four or five different portals.	HHA 6
HHA reports using lots of portals; and if not portals, waiting on phone	There's all kinds of portals that you can go into. Each Medicare Advantage plan will have their own portals. Or those that don’t, you have to call up on the phone and sit there sometimes two hours to get someone to get an authorization, but many have their own portals that you can go into and get an authorization.	HHA 13

HHA = Home Health Agency, MA = Medicare Advantage Plan, CO = Post-Acute Care Management Company.

Source: interviews with participants.

Another common approach used by MA plans to ease the burden of UM for post-acute home health care was to contract with PAC management companies and delegated providers who handle the full UM function (Theme 2b). While some plans prefer to do authorization and UM themselves, citing it being “very core” to their “overall model of care” (MA 3), other MA plan representatives noted these third parties improved the process. However, many HHA representatives report that PAC management companies and other delegated providers add another layer of review, costs, and challenges to delivering timely and efficient care without “actually doing anything other than siphon money off the top” (HH 19); conversely, some HHAs shared a more optimistic outlook on PAC management companies and their evolution (see Table 2).

Medicare Advantage plans and PAC management companies also discussed deploying—or wanting to implement—portals for submitting, reviewing, and approving materials for authorizations (Theme 2c). Medicare Advantage plan and PAC management company representatives cite that portals are designed with the goal of expediting and providing transparency and consistency to the approval processes. Medicare Advantage plan and PAC management company representatives discussed how some portals use an algorithm or criteria (vs human review) to determine appropriateness of care and additional visits. They cited these algorithms/criteria as a way to ease the burden, standardize the process, and reduce the time, error, and inconsistency believed to be associated with human review. However, other MA plans and PAC management companies do not automate this process and rather use the portals as a vehicle for sending requests to a clinical team for review. While the use of portals is common among MA plans, the design and way they are operationalized varies. Therefore, HHAs report having many different portals (some as many as 10+) and processes that they have to use in their day-to-day business with MA plans and PAC management companies, leading to increased costs and administrative burden for HHAs (see Table 2).

### Theme 3. MA plans' requirements for prior authorization and UM and their implications for post-acute home health care differ from other healthcare sectors

Through interviews with MA plan and HHA representatives, we heard about how and why prior authorization and UM are different and uniquely complicated for post-acute home health. For example, unlike many other care settings, the Medicare program requires all home health orders and plans of care—regardless of if it is paid for by traditional Medicare or MA—to be signed by an approved clinician (Theme 3a, Table 3). Medicare Advantage and HHA representatives described this requirement as providing an additional layer of authorization (ordering clinician + the MA plan) on the prior authorization process. Home health agencies and Medicare Advantage plan representatives both shared that the clinician authorization requirement often complicates and delays the process of delivering care to patients, particularly if the plan also requires prior authorization: “….That's really the biggest problem that doctors have their own practices to run and home care is usually not a priority to them and getting them to pay attention to what we send them or even have it done correctly is very difficult” (HH 9).

**Table 3.** Theme 3. MA plans' requirements for prior authorization and utilization management and their implications for the delivery of post-acute home health care differ from other sectors of healthcare

Theme 3a. Doctors add another layer to the authorization process, often complicating and slowing down the approval		
MA plans report that physicians have a role separate from the plan's prior authorization process	They get the order from the physician to ensure that it will meet the needs of the patient… then that agency then starts following the patient, and goes and sees the patient, and then develops a plan of care for that member, and then that gets sent back to the ordering physician, and then they approve the plan of care with the member, and that's kind of the agency takes over after that.	MA 7
HHAs find prior authorization frustrating because a third-party physician is the person who ordered it	There is an independent third party out there, the physician, ordering the service. It's not like I’m going out there saying, “You have six wounds and I got to…” I have a physician's order telling me what to do. And some insurance companies don’t think that's enough.	HHA 9
MA plans report that doctors complicate approval process by referring to wrong HHA	And let me just clarify something that we frown upon quite a bit, there’ll be a doc who will say, “Hey, [name of agency], I’ve got a patient who has [name of MA plan] insurance. They’d be a great patient for you to pick up.” And then [name of agency] will jump in and try to take that patient, but [name of agency] may not be our priority one for that patient. And we will promptly and happily deny that service. Okay? And that's a faux pas on the physician… you better believe we’re going to go tell that physician, “Don’t do that again.”…We will offer a different option, like a different company. We don’t deny it for the service. We deny it for the choice of company because some companies are better at some things… I don’t want somebody who does home PT/OT, who dabbles in respiratory work, to get my respiratory business… everybody wants to do everything. They’re like, “We’re full service.” Well, that doesn’t work for me.	MA 5
HHA reports that doctors are a problem in authorization	One of the challenge home health has is getting a hold of the PCP for updated orders or getting those orders signed, that's always a challenge because especially patients coming from the hospital, the PCP may not be involved and some of these PCPs have not seen patients for years, and so it's just a little more challenging in working with the primary care physicians versus working with these nurse practitioners who sees the patient timely.	HHA 16
HHA reports that doctors are a problem in authorization; limits patient access	Doctors totally ignoring the orders. That's really the biggest problem. Doctors have their own practices to run and home care is usually not a priority to them and getting them to pay attention to what we send them or even have it done correctly is very difficult…. There are some doctors, believe it or not, whose patients we will not take because they never fill out the paperwork.	HHA 9

Theme 3b. Home health is episode-based; but there is no standard for what is considered an episode or unit of service across MA plans		
HHA reports variation in episodes and units of care by plan	It's different for every plan pretty much. There's really no standard. I think the standard is that home health episodes run in 60-day episodes, but within it there's 30-day periods. For Medicare, you’re going to build your plan based on the 60 days. However, you may not go all 60 days or you might re-certify more. Some of our episodic MA plans runs very similar… We also have MA plans that will say, “Here's two visits. Send us your documentation and we’ll give you two more,” and so it's hand over fist, just always asking for authorization.	HHA 6
HHA reports variation in approvals, by payer	When you have the particular payers that are maybe PPO, they pay you by episode. Once you get approved for all the services, you pretty much have your approval up front. But with the other payers, it is per visit type of thing, then some of the insurances are actually, even after they’ve approved your visits, if they call for maybe your documentation or your chart, they review it and they say that, “Medical necessity was not met, and we’re not going to pay.” …. The HMOs or the episodic payers really do not put a limit on how many visits you can order…[Name of plans], they’ll tell you, after they get the information, the initial assessment and the plan of care, they will review it and say how many visits you’re approved for. Every two weeks, we’re having to get authorization for them to review.	HHA 3
HHA reports that the process and number of approved visits vary by payer	And then once we send in the assessment, we’ll have to get authorizations for whatever we want for the follow-up plan, X number of visits. We will generally request a lump sum number of in-home visits from nursing, physical therapy, occupational speech, whatever it is, depending on the payer. Some payers are just very rubber stamp. You get 10OT, 10PT, 10 nurse, 10 whatever, come back to us after you do this. Some are the exact opposite where they’ll be you have two nursing visits, after you complete those and only after you’re done send us the documentation of the two visits, send us your follow-up plans.	HHA 5
HHA reports that some plans pay by visit and some pay episodic/flat rates	And so sometimes it's week by week getting those visits authorized so we can schedule them out… there are some that pay us per visit on our visits and they pay a flat rate no matter what the discipline is. So, I might have a reimbursement that pays X amount, and it's the same whether it's a physical therapist, a physical therapy assistant, an RN or an LPN… Some of the Medicare Advantage plans are also episodic payers, and so they follow the Medicare model. And then we have another of Medicare Advantage plan that we’ve worked with in the past that paid us a case rate, which was flat rate. They didn’t care what the patient's comorbidities were, didn’t matter what the diagnosis was, didn’t matter how acute they were, they would pay us to stay flat rate. And then after for 30 days, and then after those 30 days when we would request authorization for the following 30 days, it was very, very challenging to get authorization. And so often we would be stuck with patient in the middle of an episode that we couldn’t do a discharge visit on.	HHA 7
HHA reports that patients switching MA plans during an episode compromises ability to get paid	One of the biggest problems in healthcare is people switching and providers not being notified. We used to check our eligibility and their insurances twice a month, the 1st and the 15th. It's gotten so bad. [Name of Duals plan] is a disaster. We’ve had a computer program written for us that checks it every single day now. Because the changes are dramatic, and if we miss that, and we don’t get the authorization, we’re not getting paid.	HHA 9
HHA reports that patients switching MA plans changes care provided and need to get re-established	“It becomes a burden when in the midst of an episodic episode such as a patient was admitted under traditional Medicare, well open enrollment started and this patient is still receiving care from us… It's like going through an admission process all over again because the patient didn’t really know, unbeknownst to them that this changes everything for them. They may not be able to have three days a week. Now because they made the switch not because of punishment, but because of the insurance inability to cover all three visits, it becomes only two days, and we have to explain that. So that becomes the burden for us when a patient is already established and has to go through that process all over again to get re-established.”	HHA 17
**Theme 3c. Home health agencies are challenged with adhering to regulations related to timeliness of care—and exposed to financial risks—because of prior authorization requirements**		
HHA must provide care because of regulatory standards; financial risks to HHA for seeing patient without prior authorization	Sometimes you get what you ask for, sometimes you don’t. That's a very difficult process as well, because once a home care agency accepts that patient on service, they are by law, by Medicare requirements, required to provide the care that patient needs…The care can’t even be scheduled in some of the electronic medical records systems with home care until there's auth, but yet we have a patient who is requiring this care. We accept it on service. They have a managed care provider who takes three to six days, maybe a week to get back regarding additional auth. So, they are required because of their regulatory standards to provide care for that patient, even if they don’t have auth, which oftentimes they’re not retro-activated.	HHA 8
HHA must provide timely care because of Medicare's condition of participation requirements; but because of financial implications is delaying services	Authorization is a big issue… We’ll get the referral from a hospital, then we have to go to a Medicare Advantage plan and get authorization for visits. The patient, a hospital may want you to see, and the doctor may want you to see the patient 24 to 48 hours from the time that the patient's discharged, which is a Medicare condition of participation, so it's a guideline. We may not get authorization for 14 days from the Medicare Advantage plan…We were writing off way too much money each year because of the authorization process. And our people that were doing the auth process would just push through the visit to scheduling, tell them, “Go ahead and do the visit,” before we would get authorization because they were feeling pressure from the branches. And we found that out the hard way… So, we’re holding up visits now, and we’re telling the patients, we’re telling the referral sources, “It's not us.” And if we have to delay service, we’re going to delay service.	HHA 13
HHA will see patients pro bono to start care while looking for another agency	We had called around to some different other home care providers in our area, found one that could take the patient, but not for two more days. We had agreed as an agency, “Okay, we’re going to see them today when they get home, we’ll see them tomorrow for the follow-up and then agency B has agreed to pick them up, let's say, on Friday to continue services.” We have done these things pro bono if needed to avoid abandoning the patient once we accept them.	HHA 1
HHA's culture is to see patient even without authorization	The culture of this particular organization is we don’t stop seeing a patient just because we don’t have an auth. We still give them 72 hours' notice and let them know that this is the case… they can call their doctor, they can call their insurance company, but in 72 hours, we’re going to have to move forward to a different solution for you. But we don’t just drop them right off.	HHA 11
HHA's mission is to never say no	We never say, “No, we’re not going to see you until we get your authorization,” because it's critical timing for a patient, those first 14 days, 20 days, but we run a risk of a lot of write-offs if that comes back denied for some reason.	HHA 6

HHA = Home Health Agency, MA = Medicare Advantage Plan, CO = Post-Acute Care Management Company.

Source: interviews with participants.

In addition, the episode-based nature of HHA creates challenges for prior authorizations and UM (Theme 3b). Unlike traditional Medicare where episodes (and payment) are based on 30 and 60 days, MA plans have discretion about whether they pay for services on an episodic basis and what they define as an episode. Therefore, HHAs report that prior authorizations are for varying amounts of care and complicate care planning. In addition, HHAs report that delivering care to patients in MA plans with episodic payments is particularly complicated because of patients' frequent switching among MA plans and traditional Medicare.

Home health agencies also shared how prior authorization requirements challenge their ability to adhere to regulations related to timeliness of care and expose them to financial risks (Theme 3c). While some HHAs said that they would provide care to patients referred to their agency without prior approval from MA plans—posing financial risk to an agency should that approval later be denied—other agencies reported waiting to see patients until authorization was granted running counter to their care philosophy and regulatory requirements while also compromising patient care (see Table 3).

### Theme 4. Home health agencies report that prior authorization and UM requirements impact access to care, the way that care is delivered, and patients' experiences

Home health agencies shared many examples of the ways in which MA plans' prior authorization and UM approaches impact the plans with which they contract, the patients whose referrals they accept, the way that they deliver care, and patients' experiences and outcomes (see Table 4). We heard from HHA representatives that frustrations with authorization and UM requirements have led to restricting which and how many plans with which they contract. Home health agencies also described how prior authorization and UM requirements impact their decisions on which patient referrals to accept, with many HHAs saying that they will not accept referrals for patients from MA plans with burdensome administrative requirements.

**Table 4.** Theme 4.

Theme 4. Home health agencies report that prior authorization and UM requirements impact access to care, the way that care is delivered, and patients' experiences		
HHA considers UM process when deciding to accept patients	[In response to the question: “so what does your organization look for in an MA plan when deciding if you want to be an in-network provider?”] I think the big determining factor…their utilization review processes. How onerous is their UM processes, and do they have a lot of pre-cert requirements?… how difficult are they to deal with to get the requirements?	HHA 18
HHA limits number of plans they work with	We work mainly with about three managed care plans, just because it's much easier… We understand their process, they understand our process.	HHA 16
HHA shares that the patients are delayed in being discharged from hospital because of the authorization process	There are delays in discharge due to the auth process with managed care plans, and in some hospitals, it will cause several days' delay in discharge from the hospital, waiting for that managed care plan to either approve or deny the home care request… Very common, happens every single day, multiple times a day… These plans are adding to increased length of stay for these patients that are ready to be discharged from the hospital.	HHA 8
HHA delays service until prior authorization is received	There are delays in starting care because we have not gotten authorization yet. We get the referral; we send over the authorization right away… We just wait until we get auth, and so I watch the timeliness get longer and longer from discharge to admission, and I worry about the little ladies and gentlemen that we’re caring for and so that's a little bit frustrating, ‘cause it takes some time…the whole system is a lot of bureaucratic waste really.	HHA 16
HHA reports that prior authorization challenges clinicians' abilities to treat patients	Our plan of care may call for 15 visits in a 60-day episode. They may only give us authorization for six visits and then we’ve got to go back and get reauthorization. So, they put such a burden on our clinicians' ability to treat the patient the way they need to be treated.	HHA 13
HHA reports that plans don’t authorize home health for patients who need it	They don’t give authorization… So [Name of Plan] is only limiting three PT and one nursing. I mean, it's insane. We’re talking hours of work and then they’ll deny it. It's like, what are you denying? The patient can’t even function. And they’re saying no, based on they can walk, I don’t know, 50, 100 feet. Well, then they’re not homebound. Well, that's not true. If it's taxing effort, they are homebound and they’re at risk for falling. It just goes crazy.	HHA 2
HHA describes patients waiting at home while agency is jumping through hoops	I think the thing we’ve been talking about recently and I’ve been trying to remind myself and us is somebody's mother is sitting at home waiting for care. Let's just start right there. And then the reason I say that is because there's so many hoops you have to jump through. We do find that ourselves, and I think lots of others getting caught up in the hoop and forgetting that somebody's sitting at home waiting for care and that first week, the two weeks is really the highest risk in terms of readmission.	HHA 5
HHA describes that patient is at home waiting in pain while they wait for approval	The problem is the patient suffers because the patient's at home waiting and in pain or needs something and we can’t get out there because they haven’t approved us yet, so it's that kind of stuff. If we didn’t have these things, we could go out same day, next day and get it going, but we can’t.	HHA 9

HHA = Home Health Agency, MA = Medicare Advantage Plan, CO = Post-Acute Care Management Company.

Source: interviews with participants.

Home health agencies provided several examples about patients in need of post-acute home health care spending longer in the hospital, or longer at home without care “waiting and in pain” (HH 9), because of a prior authorization requirement. This phenomenon was described as frequent and an “issue” they “deal with every day” (HH 4). Finally, HHA representatives discussed examples of how limitations on visits impact patients' outcomes. One HHA representative shared: “I can see it clearly in outcomes with the payers that are authorized vs not authorized… they have a measurably lower number of visits to home health, and they have lower quality outcomes… It's simple math” (HH 19).

## Discussion

Interviews with 44 representatives from MA plans, PAC management companies, and HHAs highlighted the motives, players, processes, challenges, and impacts of prior authorizations for post-acute home health care. Participants revealed variations in rationales and approaches, the complexities of securing approvals, and the unique difficulties of prior authorization and UM compared to other sectors. Home health agency representatives also described how UM requirements from MA plans and PAC management companies compromised the care they provided.

Prior authorizations in MA vary by service type, cost, potential overuse, and medical complexity, with high-cost and specialized services often requiring authorization.^16,17^ Despite being a lower-cost PAC option and MA plan representatives report preferring that patients receive care in their homes, home health frequently requires prior authorizations.^18^ Medicare Advantage plan representatives note that requiring prior authorization ensures that patients with a true need for PAC home health receive it, while simultaneously being useful for controlling costs and weeding out fraudulent or unnecessary claims by “bad actors”. Interviewees noted that authorization processes differ across plans and are administratively burdensome for both MA plans and HHAs. Many MA plans delegate these functions to third-party providers and PAC management companies, which, while intended to reduce administrative strain for MA plans, often add complexity to UM processes for HHAs. The role of PAC management companies and delegated providers in PAC access, specifically care provided in skilled nursing facilities, faces growing scrutiny as highlighted by the 2024 Senate report^19^ and recent news articles.^20^ Further research is needed to evaluate how these third parties affect MA beneficiaries' experiences and outcomes.

Home health agency representatives shared that MA plans and PAC management companies' prior authorization requirements demand extra resources, such as staff, creating unreimbursed costs that strain an already financially vulnerable industry,^21^ particularly post-COVID^22-24^ and amid recent regulatory changes. Of note, in March of 2024, MedPAC recommended lowering home health payments in traditional Medicare due to concerns about overpayment.^25^ This requires substantial consideration, given the way policies and payment models in traditional Medicare often spill over into MA and the already considerable challenges our participants identified in working with MA. To meet their mission and adhere to Medicare regulations to complete an initial assessment within 48 h of referral or a patient's return home (42 CFR 484.55), HHAs often provide care without prior authorization, risking further financial instability if services aren’t approved or reimbursed. Future research should examine the costs and risks to HHAs of MA plans' prior authorization requirements and their impact on the industry's viability.

Participants highlighted the use of portals for UM and the variability in human vs algorithmic reviews. A Senate report noted the increasing reliance on algorithms for PAC decisions,^19^ while a 2022 OIG report found that 1 in 5 denied MA payment requests should have been approved, citing human and system processing errors.^26^ With about 1.5 million prior authorizations denied in 2018, our findings, along with these reports, suggest no single optimal approach but underscore the need for greater oversight and auditing of these processes.

Our qualitative interviews add rich understanding to the literature; while prior quantitative research has reported that MA plan beneficiaries receive less home health care (eg, fewer referrals to home health and fewer home health days) compared to their traditional Medicare counterparts,^8,9^ the literature differs on whether MA is associated with better or worse outcomes for PAC home health beneficiaries.^11,25^ Our findings highlight substantial variation in the motivations and approaches used by MA plans to carry out prior authorizations for PAC home health. This variation may help to explain prior quantitative inconsistencies. Interviews with HHA representatives revealed that prior authorization delays care, compromises access, and affects patient outcomes, aligning with prior research on the administrative burdens of payers.^27,28^ Some HHAs reported limiting contracts or refusing referrals from MA plans with onerous UM requirements, restricting patient access to home health. These findings may explain earlier research showing MA plans often contract with lower-quality HHAs,^29^ who may be less able to pick and choose the plans with which they contract. Given documented disparities in home health access for MA enrollees, as well as nonwhite, rural, or segregated populations,^10,30-36^ future research is needed to explore the potential dual disparities affecting these groups and their impact on health equity.

Our findings on prior authorization delays align with those from other settings. The American Medical Association reported that 91% of physicians experienced care delays due to prior authorization, while a Kaiser Family Foundation study^37^ found MA enrollees in plans requiring prior authorization were 3 times more likely to face delays and twice as likely to report health decline due to those delays. Given that post-discharge home health patients are clinically complex and vulnerable,^38,39^ timely care is crucial for positive outcomes.^33,40-43^ Our results support the importance of CMS's 2024 Final Rule,^44^ which aims to streamline prior authorization, improve transparency, and mandate real-time electronic systems.

Our findings suggest the need for further investigation into prior authorization processes for home health care. While the OIG is investigating prior authorization in PAC,^7^ it currently only focuses on long-term acute care hospitals, inpatient rehabilitation facilities, and skilled nursing facilities. Given that HHA representatives report MA plans' prior authorization processes delay services and compromise patient care, home health should also be included in these investigations. Additionally, publicly reporting denial information for post-acute home health care, as is being called for in other PAC settings by the US Senate,^19^ would enhance transparency and understanding of MA's prior authorization processes.

Finally, our findings, while focused on prior authorization for post-acute home health in the MA program, have broader implications for traditional Medicare. The first surrounds the challenges with seeking ordering clinician approval for initiation of home health services that were expressed by MA plan and HHA representatives, alike. These challenges and delays associated with the need to seek an additional layer of approval are echoed in prior research not specific to MA.^45^ While the CARES Act passed in 2020 allowed for a nurse practitioner, clinical nurse specialist, or a physician assistant who is working in accordance with state law to order and certify home health care, we still heard about the challenge obtaining this approval even after this expansion in the types of clinicians who can order home health became permanent at the end of the public health emergency (and codified in the regulations at 42 CFR 409.43). These findings suggest that consideration needs to be given around the Medicare home health benefit requirement for clinician review and approval prior to initiating home health services, possibly through legislation that allows for historically compliant providers to provide the care without additional need for clinician documentation or approval.

## Conclusion

In conclusion, our findings suggest that prior authorization is an administrative burden for both MA plans and HHAs. Despite efforts to ease this burden, most MA plans still require prior authorization for post-acute home health care. Home health agency representatives raised concerns about how prior authorization and UM requirements compromise patient access and care. These findings call for further research and policy attention to ensure that UM practices by MA plans do not unintentionally harm vulnerable patients in need of post-acute home health care.

## Supplementary Material

qxaf020_Supplementary_Data
