# Supplementary material for: Prior authorization and utilization management for post-acute home health in Medicare Advantage: the motivations, players, processes, unique challenges, and impacts on patient care
Source: Health Aff Sch. 2025 Feb 4;3(3):qxaf020. doi: 10.1093/haschl/qxaf020 (PMC11886789; doi:10.1093/haschl/qxaf020)
Supplement: qxaf020_Supplementary_Data [file qxaf020_supplementary_data.zip › PriorAuth_OnlineAppendix_2.docx]

ONLINE APPENDIX

APPENDIX Table 1
Caption: Appendix Table 1. Characteristics of Medicare Advantage Plans Participating in Interviews
Source/Notes:

SOURCE: Authors’ summary of interviewed Medicare Advantage plans using 2023 data from the Centers for Medicare & Medicaid Services including Medicare Advantage Contract Summary data, Medicare Advantage Enrollment by Contract data, and Medicare Advantage Part D Star Ratings data.

NOTES: *The contract start time corresponds to the periods before the Medicare Modernization Act (before 2006), between the Medicare Modernization Act and the launch of the Medicare Advantage Quality Improvement Program (2006-2013), and between the Patient Protection and Affordable Care Act and the current year.

Average star ratings represent the average of all of the plans’ star ratings within a contract in 2023.

Total enrollment is the total number of Medicare beneficiaries enrolled in the contract in December 2023.

APPENDIX Table 2
Caption: Appendix Table 2. Characteristics of Home Health Agencies Participating in Interviews
Source/Notes:

SOURCE: Authors’ summary of interviewed home health agencies using 2024 Home Health Provider file from the Centers for Medicare & Medicaid Services to identify the CCN and Quality of Patient Care Star Rating, age, and scope. Authors linked home health agency CCNs to 2020 OASIS assessment data to identify the number of unique start of care assessments.

NOTES: "Average star rating" is represented by averaging Q1 2024 Quality of Patient Care Star Ratings, which is reported by CCN, across all CCNs belonging to a home health agency.

Start of care assessments are the number of unique patients with a start of care assessment in CY2020.

**Table 1**. Characteristics of Medicare Advantage Plans Participating in Interviews

| ID | Plan Scope | Average Star Rating | Contract Start Date* | Total Enrollees |
| --- | --- | --- | --- | --- |
| MA 1 | Regional | 4.6 | Before 2006 | 100,000-500,000 |
| MA 2 | Regional | 4.7 | Before 2006 | 100,000-500,000 |
| MA 3 | Regional | 4.5 | Before 2006 | <100,000 |
| MA 4 | Regional | 5 | Before 2006 | <100,000 |
| MA 5 | National | 2.9 | Before 2006 | >1,000,000 |
| MA 6 | Regional | 5 | Before 2006 | <100,000 |
| MA 7 | National | 4.8 | Before 2006 | >1,000,000 |
| MA 8 | National | 3.9 | Before 2006 | >5,000,000 |
| MA 9 | Regional | 3.7 | Before 2006 | 100,000-500,000 |
| MA 10 | National | 4 | Before 2006 | >5,000,000 |
| MA 11 | Regional | 2.5 | 2014-2024 | <100,000 |
| MA 12 | Regional | 4 | Before 2006 | 100,000-500,000 |
| MA 13 | Regional | 3.5 | Before 2006 | 100,000-500,000 |
| MA 14 | Regional | NA | 2014-2024 | <100,000 |

Notes.

*The contract start time corresponds to the periods before the Medicare Modernization Act (before 2006), between the Medicare Modernization Act and the launch of the Medicare Advantage Quality Improvement Program (2006-2013), and between the Patient Protection and Affordable Care Act and the current year.

Average star ratings represent the average of all of the plans’ star ratings within a contract in 2023.

Total enrollment is the total number of Medicare beneficiaries enrolled in the contract in December 2023.

MA = Medicare Advantage Plan

**Table 2**. Characteristics of Home Health Agencies Participating in Interviews

| ID | Agency Scope | Average Star Rating | Age in Years | Start of Care Assessments |
| --- | --- | --- | --- | --- |
| HHA 1 | Regional | 2.5 | 40+ | 10,000-100,000 |
| HHA 2 | Regional | 4 | 1-20 | <10,000 |
| HHA 3 | National | 4.3 | 40+ | >100,000 |
| HHA 4 | Regional | 3.5 | 20-40 | <10,000 |
| HHA 5 | National | 4.0 | 40+ | >100,000 |
| HHA 6 | Regional | 3.5 | 1-20 | 10,000-100,000 |
| HHA 7 | Regional | 4.3 | 20-40 | 10,000-100,000 |
| HHA 8 | Regional | 4.5 | 1-20 | <10.000 |
| HHA 9 | Regional | 4 | 20-40 | <10,000 |
| HHA 10 | Regional | 2.5 | 40+ | <10,000 |
| HHA 11 | Regional | 4.1 | 20-40 | 10,000-100,000 |
| HHA 12 | Regional | 3 | 20-40 | 10,000-100,000 |
| HHA 13 | Regional | Too small to report | 0-20 | <10,000 |
| HHA 14 | Regional | 2.5 | 40+ | <10,000 |
| HHA 15 | Regional | 4 | 1-20 | <10,000 |
| HHA 16 | Regional | 4 | 1-20 | <10,000 |
| HHA 17 | Regional | 3.6 | 20-40 | <10,000 |
| HHA 18 | National | 4.5 | 40+ | >100,000 |
| HHA 19 | Regional | 3.5 | 40+ | 10,000-100,000 |

Notes. "Average star rating" is represented by averaging Q1 2024 Quality of Patient Care Star Ratings, which is reported by CCN, across all CCNs belonging to a home health agency

Start of care assessments are the number of unique patients with a start of care assessment in CY2020.

HHA = Home Health Agency
